# Photonic thermal management of coloured objects

**DOI:** 10.1038/s41467-018-06535-0

**Published:** 2018-10-12

**Authors:** Wei Li, Yu Shi, Zhen Chen, Shanhui Fan

**Affiliations:** 1 Department of Electrical Engineering, Ginzton Laboratory, Stanford University, Stanford, CA 94305 USA; 2 School of Mechanical Engineering, Southeast University, Nanjing, 210096 China

## Abstract

The colours of outdoor structures, such as automobiles, buildings and clothing, are typically chosen for functional or aesthetic reasons. With a chosen colour, however, one must control the radiative thermal load for heating or cooling purposes. Here we provide a comprehensive calculation of the tunable range of radiative thermal load for all colours. The range exceeds 680 Wm^−2^ for all colours, and can be as high as 866 Wm^−2^, resulting from effects of metamerism, infrared solar absorption and radiative cooling. We experimentally demonstrate that two photonic structures with the same pink colour can have their temperatures differ by 47.6 °C under sunlight. These structures are over 20 °C either cooler or hotter than a commercial paint with a comparable colour. Furthermore, the hotter pink structure is 10 °C hotter than a commercial black paint. These results elucidate the fundamental potentials of photonic thermal management for coloured objects.

## Abstract (web summary)

Understanding the tunable range of radiative thermal load for a given colour is important for thermal management of outdoor structures. Here, the authors theoretically and experimentally highlighted all mechanisms through which one can control the radiative thermal load of coloured objects.

## Introduction

Controlling the radiative thermal load of an outdoor structure, such as a building or an automobile, is essential for its energy efficiency. The radiative thermal load of a structure results from the heating of such structure by external radiation such as the sun, as well as the cooling of such structure due to heat dissipation through thermal radiation. Nowadays, heating and cooling account for about 48% of the energy use in U.S. homes, making it the largest expense for energy consumption^1^. For automobiles, the need for air-conditioning can reduce the fuel economy by 20%^2^. In addition, the health and economic threats of outdoor heat and cold stress to the daily life as well as occupational, sport and military sectors are becoming more intense and frequent^3^. Therefore, the ability of controlling radiative thermal load is of general importance for global energy consumption and human health.

In recent years there has been significant progress in designing photonic structures to control both solar absorption and thermal emission^4–19^. To minimize thermal load, for example, one can use a near-perfect mirror to reflect all the sunlight while maximizing thermal emission^13^. Alternatively, to maximize thermal load, one can use a black object that absorbs all the sunlight, but has minimal thermal radiation^14,18^. However, the colour of outdoor structures, are usually chosen first for functional or aesthetic reasons. The structures as discussed above therefore are not directly applicable for controlling the thermal load of outdoor structures since they are either perfectly reflecting^13^ or completely black^14,18^.

For common coloured objects, for example conventional paints or textiles, there has been a direct association between colour and thermal load, dating back to Benjamin Franklin’s early colour-heat experiment^20^ around 1730 s. As an example of such association, conventional paint with a darker colour tends to get hotter under the sun as compared with a paint with lighter colours. More recently, there has been a large body of work on colour objects seeking to achieve thermal loads that are different from conventional paints. For this purpose, significant efforts have been made to control the absorption of the near infrared part of the solar radiation that contributes to the thermal load but not the colour of an object^21–23^. There are also efforts to exploit radiative cooling^24^, and to control colour and thermal load in the visible part of solar spectrum using structural colour^21,22,25–27^. However, the theoretical limit over which the radiative thermal load of an object with a specific colour can vary, taking advantages of all available mechanisms for controlling thermal radiation and solar absorption, has not been explicitly considered. Here we show that for every given colour, there is in fact a very significant tunable range in its thermal load, due to the physical effects of infrared solar absorption and radiative cooling, as well as the physiological effect of metamerism, since colour arises from the human perception of the visible spectrum.

## Results

### Theoretical analysis

Consider an object with a specific colour, in an outdoor environment facing the sky, and subjects to direct sunlight. To start we first consider the radiative thermal load, the contribution to the thermal load from solar and thermal radiations only. We will treat the non-radiative contribution to the thermal load later in the paper. The net radiative thermal load of the object in general can be written as:

1 $$P_{{\rm{net}}} = P_{{\rm{sun}}}^{{\rm{visible}}} + P_{{\rm{sun}}}^{{\rm{infrared}}} - P_{{\rm{cooling}}}$$

where

2 $$P_{{\rm{sun}}}^{{\rm{visible}}} = {\int_{0.3\upmu{{\rm{m}}}}^{0.76\upmu{{\rm{m}}}}} {\mathrm{d}\lambda \cdot I_{{\rm{AM1}}.5}\left( \lambda \right) \cdot \varepsilon \left( \lambda \right)}$$

is the absorbed power density from the visible and ultra violet part of the solar spectrum; *I*_AM1.5_(*λ*) is the AM1.5 spectrum, *ε*(*λ*) is the spectral absorptivity of the outdoor object;

3 $$P_{{\rm{sun}}}^{{\rm{infrared}}} = {\int_{0.76\upmu{{\rm{m}}}}^{4\upmu{{\rm{m}}}}} {\mathrm{d}\lambda \cdot I_{{\mathrm{AM}}1.5}\left( \lambda \right) \cdot \varepsilon \left( \lambda \right)}$$

is the absorbed power density from the infrared part of the solar spectrum;

4 $$P_{{\rm{cooling}}} = P_{{\rm{rad}}} - P_{{\rm{atm}}}$$

is the net radiative cooling power in the absence of sunlight. *P*_rad_ is the total thermal radiation power by the object and can be calculated as

5 $$P_{{\rm{rad}}} = {\int} {\mathrm{d}{\mathrm{\Omega }} \cdot {\mathrm{cos}}\theta {\int_0^\infty} {\mathrm{d}\lambda \cdot I_{{\rm{BB}}}(} } T,\lambda ) \cdot \varepsilon (\lambda ,{\mathrm{\Omega }})$$

where${\int} {\mathrm{d}\Omega = {\int}_0^{\pi /2} {\mathrm{d}\theta \sin \theta {\int}_0^{2\pi } {\mathrm{d}\phi } } }$ is the angular integral over the hemisphere. $I_{\mathrm{BB}}\left( {T,\lambda } \right) = \left( {2hc^2/\lambda ^5} \right)/\left[ {e^{hc/\lambda k_{\mathrm{B}}T} - 1} \right]$ is the spectral radiance of a blackbody at temperature *T*, where *h* is the Planck’s constant, *c* is the velocity of light, *k*_B_ is the Boltzmann constant. *P*_atm_ is the absorbed thermal emission power from the atmosphere at ambient temperature *T*_amb_ and can be calculated as

6 $$P_{{\rm{atm}}} = {\int} {\mathrm{d}{\mathrm{\Omega }} \cdot {\mathrm{cos}}\theta {\int_0^\infty} {\mathrm{d}\lambda \cdot I_{{\rm{BB}}}(} } T_{{\rm{amb}}},\lambda ) \cdot \varepsilon (\lambda ,\Omega ) \cdot \varepsilon {}_{{\rm{atm}}}(\lambda ,{\mathrm{\Omega }})$$

where $\varepsilon {}_{\mathrm{atm}}(\lambda ,\Omega ) = 1 - t(\lambda )^{1/\cos \theta }$ is the angle-dependent emissivity of atmosphere and *t*(*λ*) is the atmosphere’s transmittance in the zenith direction.

**Fig. 1:**
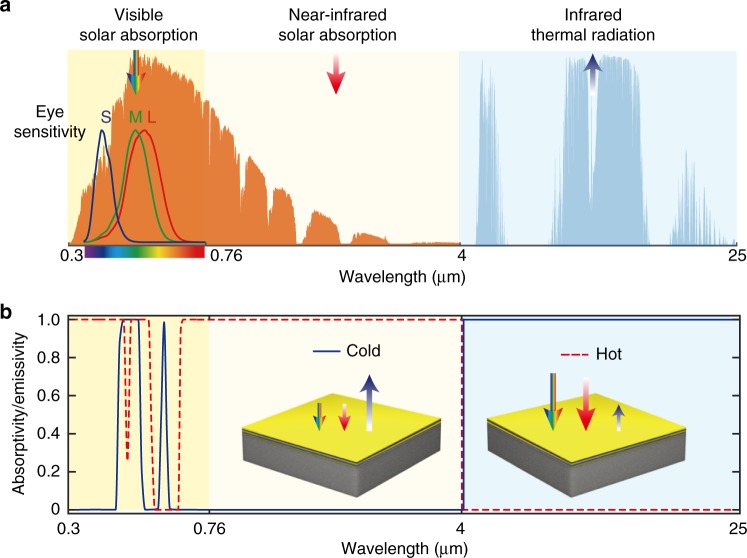
Mechanisms for photonic thermal management of a coloured object. **a** Three important mechanisms: visible solar absorption, near infrared solar absorption and infrared thermal emission for colour-preserving photonic thermal management in the visible, near infrared and thermal wavelength range. The human eye sensitivity spectra of three receptors with short ‘S’ (blue curve), middle ‘M’ (green curve) and long ‘L’ (red curve) sensitivities^29^, AM 1.5 G solar spectrum (orange shaded area), and atmosphere transmittance^28^ (light blue shaded area) are plotted for references. **b** Exemplary absorptivity/emissivity spectra of two surfaces with the same ‘gold’ colour but significantly different thermal properties. The ‘cold’ surface (blue curve, left inset) has high reflection/low absorption in visible and near infrared wavelength range, as well as high thermal emission in the wavelength range. The ‘hot’ surface (dashed red curve, right inset) has low reflection/high absorption in visible and near infrared wavelength range, as well as low thermal emission in the thermal wavelength range

From Eq. (1), objects that have the same colour response in an outdoor environment can have drastically different thermal loads, because each of the terms in Eq. (1) can have a substantial range of variability (Fig. 1a):

1. $P_{{\rm{sun}}}^{{\rm{infrared}}}$: The infrared portion of the solar spectrum does not contribute to the colour property of object. There is, nevertheless, a significant part of the solar power in the wavelength range from 0.76 μm to 4 μm. For AM 1.5 spectrum, $P_{{\rm{sun}}}^{{\rm{infrared}}}$ can vary from 0 Wm^−2^ to 453 Wm^−2^, depending on the solar absorptivity of the object in this wavelength range.
2. *P*_cooling_: In the infrared wavelength range between 4 μm and 25 μm, there is a lack of solar energy. On the other hand, an outdoor object facing the sky can emit heat out to the outer space by thermal radiation through the atmosphere transparency window between 8 μm and 13 μm. Such a process, known as radiative cooling^4–6,13^, does not affect the colour but contributes to a negative thermal load, or radiative cooling power *P*_cooling_. At 298 K, assuming an atmospheric transmission spectrum of a clear day in California^28^, and depending on the object’s infrared emissivity spectrum, *P*_cooling_ can vary between 0 Wm^−2^ and 130 Wm^−2^.
3. $P_{{\rm{sun}}}^{{\rm{visible}}}$: In the visible and ultra violet part of solar spectrum from 0.3 μm to 0.76 μm, the spectral absorption and reflection properties of the object dictate both the colour and $P_{{\rm{sun}}}^{{\rm{visible}}}$. However, the human eye contains only three colour receptors with sensitivity span in the visible spectrum^29^ (Fig. 1a). Thus for colour vision, all spectra are reduced to three tristimulus values^30^. As a result, light with different spectral power distributions may nevertheless produce an equivalent receptor response and colour sensation. This physiological effect is known as metamerism^30^. Therefore, two surfaces that have very different solar absorption in the visible wavelength range, and hence very different $P_{{\rm{sun}}}^{{\rm{visible}}}$, may appear indistinguishable in colour. We note the range of thermal load induced by such physiological effect in controlling the thermal load of outdoor structure has not been previously computed explicitly. As an example, in Fig. 1b, both absorptivity spectra produce a gold colour as shown in the insets. But their visible solar absorption power $P_{{\rm{sun}}}^{{\rm{visible}}}$, at 125.5 Wm^−2^ and 376.5 Wm^−2^, respectively, differs by about a factor of 3.

**Fig. 2:**
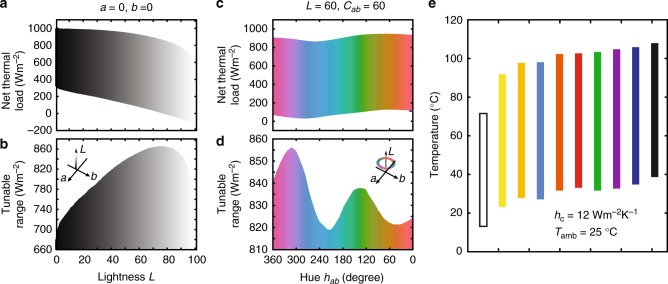
Thermal management potential of colours. **a**, **b** Net radiative thermal load (**a**) and tunable range (**b**) of colours with fixed chromaticity (*a* = 0, *b* = 0), as a function of lightness (*L* from 0 to 100). The inset in Fig. 2b indicates the location of the computed colours in 3D colour space. **c**, **d** Net radiative thermal load (**c**) and tunable range (**d**) of colours with fixed lightness (*L* = 60) and saturation (*C*_*ab*_ = 60), as a function of hue angle ($h_{{{ab}}} = \arctan \left( {b/a} \right)$ from 360° to 0°). The inset in Fig. 2d indicates the location of the computed colours in 3D colour space. **e** Tunable temperature range of commonly used colours under a typical non-radiative heat dissipation condition *h*_c_ = 12 Wm^−2^·K^−1^

Therefore, combining all three terms in Eq. (1) as discussed above, one can have objects with same colour response but significantly different net radiative thermal load. Here we consider different spectra for the same colour parameters and compute the tunable range of thermal loads using Eq. (1) for all colours (Methods). Every colour can be described numerically by three parameters labelled *L*, *a* and *b*, where ‘*L*’ represents its lightness, and the combination of ‘*a*’ and ‘*b*’ determines its chromaticity, which includes both the saturation $C_{{{ab}}} = \sqrt {a^2 + b^2}$ and the hue $h_{{{ab}}} = {\mathrm{arctan}}\left( {b/a} \right)$ of the colour^31^. As illustrations, here we provide two examples. In Fig. 2a, b, we consider grayscale colours, which have the same chromaticity (*a* = 0, *b* = 0). The colours vary from black to white as we vary the lightness from 0 to 100. For each of these grayscale colours, the minimum and maximum radiative thermal loads differ substantially. At *L* = 78, which corresponds to a light grey colour, for example, the thermal loads can vary from −3.7 Wm^−2^ to 862.3 Wm^−2^ (Fig. 2a), corresponding to a tunable range (Fig. 2b), defined as the difference between the maximum and minimum radiative thermal loads, of 866 Wm^−2^. In general, these grayscale colours exhibit a substantial tunable range varying from 680 to 866 Wm^−2^. In Fig. 2c, d, we consider a set of colours with the same lightness *L* = 60 and saturation *C*_*ab*_ = 60. As the hue varies from 360° to 0°, the colours change from purple to red. The tunable range of this set of colours varies from 820 to 856 Wm^−2^ (Fig. 2d). We have carried out the study over the entire colour space (Supplementary Note 1). The minimum tunable range occurs for both black (*L* = 0, *a* = 0, *b* = 0) and white (*L* = 100, *a* = 0, *b* = 0) at 680 Wm^−2^. The maximum tunable range occurs for the light grey as discussed above at 866 Wm^−2^. And all other colours have a tunable range in between. These results indicate that there are significant tunable range of at least 680 Wm^−2^ in radiative thermal load for every colour. This is a surprisingly large tunable range—it is in fact comparable in magnitude to the total power density of AM1.5 solar spectrum of 1000 Wm^−2^.

Based on the thermal load of the colours, we study the equilibrium temperatures for coloured surfaces under a typical outdoor condition, by balancing *P*_net_ with non-radiative heat loss *P*_cond + conv_ = *h*_c_(*T* − *T*_amb_), where *h*_c_ is the combined non-radiative heat coefficient taking into account both conduction and convection. Figure 2e, which shows the equilibrium temperatures of some commonly used colours assuming a typical *h*_c_ = 12 Wm^−2^ K^−1^ (Supplementary Note 2), reveals important aspects regarding how a colour of an object relates to its thermal properties. First, objects with the same colour can have their temperature differ by more than 70 °C in a typical outdoor condition. Moreover, an object with a lighter colour can have a much higher temperature compared to an object with a darker colour. For example, an object with a light blue colour can be over 60 °C hotter than an object with a dark blue colour (Fig. 2e). Most strikingly, a white object can be over 30 °C hotter than a black object (Fig. 2e), in contrast to the fact that a conventional black paint typically reaches the highest temperature in an outdoor environment under sunlight. Our theoretical results indicate that depending on the applications, one can construct objects with identical colours in an outdoor environment but with very different temperatures.

### Experimental design

**Fig. 3:**
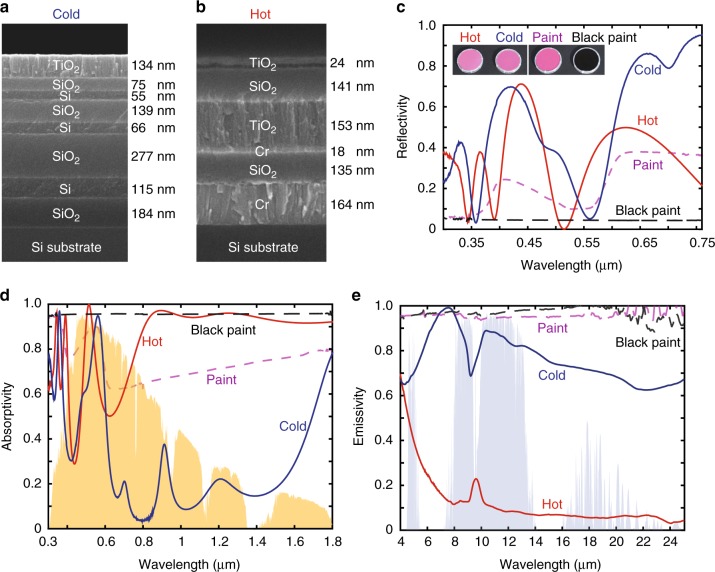
Experimental realizations of two surfaces with same colour but very different radiative thermal loads. **a**, **b** Cross-section scanning electron microscopy images of the ‘cold’ (**a**) and ‘hot’ (**b**) photonic structures, with the materials and thickness of each layer. Both of the samples are deposited on 500-μm-thick, 100-mm-diameter silicon wafers. **c** Reflectivity spectra of the ‘cold’ (blue curve) and ‘hot’ (red curve) photonic structures in the visible range demonstrating metamerism. The reflectivity spectra of a commercially available pink paint with similar colour (dashed purple curve) and a black paint (dashed black curve) are also plotted for references. Inset photos show from left to right the ‘hot’, ‘cold’, pink paint, and the black paint sample, respectively. **d** Absorptivity spectra of the ‘cold’ (blue curve) and ‘hot’ (red curve) photonic structures, the pink paint (dashed purple curve) and the black paint (dashed black curve) samples in the solar wavelength range, with the normalized AM1.5 solar spectrum plotted as the yellow shaded areas. **e** Emissivity spectra of the ‘cold’ (blue curve) and ‘hot’ (red curve) photonic structures, the pink paint (dashed purple curve) and the black paint (dashed black curve) samples over the infrared wavelength, with atmosphere transmittance^28^ plotted as the light blue shaded area for references

Based on the theoretical analyses above, we now experimentally demonstrate that objects with the same colour can have drastically different thermal response. We design and fabricate two photonic structures made of one-dimensional thin film stacks (Fig. 3a, b) that exhibit the same pink colours (inset photos in Fig. 3c) but differ significantly in their radiative thermal loads. A numerical optimization scheme is developed to design the photonic structures’ spectral properties to fulfil both the colour and thermal requirements (Methods). As shown in the scanning electron microscope cross-section image (Fig. 3a), the ‘cold’ photonic structure consists of 7 alternating layers of silicon (Si) and silicon dioxide (SiO_2_) and a layer of titanium dioxide (TiO_2_) as the top layer. The refractive index contrast between Si and SiO_2_ provides the high reflection in the near infrared region, a useful feature for minimizing the solar absorption. The combination of SiO_2_ and TiO_2_ with properly designed thickness is primarily responsible for the large thermal emissivity in the mid-infrared wavelength range for radiative cooling purpose, as both materials are emissive in this wavelength range. On the other hand, the ‘hot’ photonic structure (Fig. 3b) consists of three layers of metal-insulator-metal (MIM) on the bottom and three dielectric layers on the top. We use chromium (Cr) as the metal component in the MIM structure since Cr is highly lossy in the solar wavelength range but strongly reflective in the thermal wavelength range, therefore creating high solar absorption, yet strongly suppressing thermal emissivity. The combination of the material properties and rationally designed layer thickness further allows one to fine tune the visible reflection spectrum to achieve desired colour, while having very different thermal properties.

We compare the spectral properties of the two photonic structures in visible, solar, and mid-infrared thermal wavelengths (Fig. 3c–e). First, in the visible spectrum, the two photonic structures exhibit different reflectivity spectra, yet showing the same pink colour to the human eye (Fig. 3c), demonstrating the effect of metamerism. In the visible wavelength range (wavelength range of 0.3–0.76 μm in Fig. 3d), the solar absorption of the ‘hot’ and ‘cold’ photonic structures are 350 Wm^−2^ and 250 Wm^−2^, respectively. Our results here thus demonstrate the importance of metamerism for thermal management. Second, in the infrared part of solar spectrum (wavelength range of 0.76–1.8 μm in Fig. 3d), which does not contribute to the colour response, the solar absorption of the ‘hot’ and ‘cold’ structures are 380 Wm^−2^ and 80 Wm^−2^, respectively. Thus, for thermal management purposes, it is also important to control infrared solar absorption. Third, in the thermal wavelength range (Fig. 3e), we observe that the ‘cold’ photonic structure has a remarkably strong and broadband thermal emissivity, resulting a *P*_cooling_ of 99.7 Wm^−2^ at 298 K. In contrast, the ‘hot’ photonic structure has a strongly suppressed emissivity, with a *P*_cooling_ of only 14.2 Wm^−2^ at 298 K. As a result, the total radiative thermal loads of ‘hot’ and ‘cold’ photonic structures are 716 Wm^−2^ and 230 Wm^−2^, respectively. And all three effects above contribute significantly to the total radiative thermal load difference of 486 Wm^−2^ between these two structures.

As references, we also consider two commercially available paints coated on identical 100-mm silicon wafers (inset photos in Fig. 3c). One paint sample is with similar pink colour as the two photonic structures, whereas the other paint is black. Both the pink paint sample and the black paint sample have strong solar absorptivity yet strong thermal emissivity (Fig. 3c–e). Thus, with photonic engineering, the two photonic structures exhibit spectral properties that differ significantly from conventionally colour paints.

### Experimental results

**Fig. 4:**
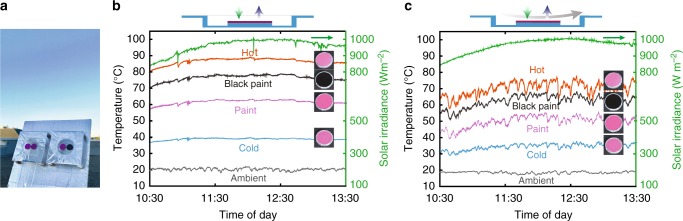
Outdoor temperature performances of two photonic structures with same colour. **a** Photo of the temperature measurement apparatus on the test rooftop in Stanford, California. **b** Rooftop temperature measurements of the ‘cold’ (blue), ‘hot’ sample (red), a pink paint with similar colour (light purple) as well as a black paint (black), ambient air temperature (light grey), and solar irradiance (green) on a clear fall day in Stanford, California. In this measurement, a low-density polyethene film covers the apparatus to reduce the air convection effect, as shown in top schematic. **c** Rooftop temperature measurements of the same samples as Fig. 4b but allowing free airflow convection, to mimic the typical outdoor condition, as shown in top schematic

We then characterize the thermal performance of the ‘cold’ and the ‘hot’ photonic structures, as well as the two paint samples in an outdoor environment (Methods). We expose all the samples to sunlight and sky by placing them on a building roof (Fig. 4a), during daylight hours on a clear fall day in Stanford, California. The experimental apparatus consists of two identical chambers placed side by side, with two photonics structures placed in the first chamber and two paint samples placed in the second chamber (Fig. 4a). Each chamber consists of acrylic walls and polystyrene supporters.

In the first experiment, to reduce the effect of wind while allowing for high transmittance of both solar and thermal radiation, we cover the opening of the chamber with 12.5-μm-thick infrared transparent polyethylene film (Supplementary Note 3). As shown in the temperature data of Fig. 4b, the two photonic structures differ significantly in temperatures. The ‘hot’ photonic structure reaches over 88 °C at noontime when the solar irradiance is over 900 Wm^−2^ and the averaged temperature for the entire 3 h measurement period is 86.5 °C. In contrast, the ‘cold’ sample constantly stays below 40 °C, with an averaged temperature of 38.9 °C. As a result, the two visually similar samples show a drastic difference of 47.6 °C in the averaged temperatures.

To further illustrate the significance of this result, we compare the thermal performance of the two photonic structures against the two paint samples. First, the averaged temperature of the pink paint sample reaches 61.5 °C. As a result, our ‘cold’ photonic structure shows a temperature that is 22.6 °C colder than the pink paint, whereas our ‘hot’ photonic structure shows a temperature that is 25 °C hotter than the pink paint. These results indicate that with photonic engineering, one can achieve similar colours as compared to conventional paints, but with drastically different thermal response that can be utilized for either heating or cooling applications. Second, our hot photonic structure with a pink colour can reach 10 °C hotter than the black paint, which is usually thought to be the hottest under sunlight. Such a result further illustrates the great potential of using photonic structures for thermal management purposes. The experimental results here can be well accounted for by the theoretical modelling that takes into account the spectral response of the samples over solar and thermal wavelengths, the non-radiative heat dissipation, the ambient air temperature, and the solar irradiance incident on the samples (Supplementary Note 3). And we have performed further experimental study to specifically analyze the effect of radiative sky access on the thermal performances of the photonic structures (Supplementary Note 4).

In the second experiment, to mimic a typical outdoor condition with substantial convection heat dissipation induced by strong wind flow, we characterize the temperatures of all samples with the same apparatus but remove the polyethylene cover. As shown in Fig. 4c, all of the four samples show lower temperatures than their covered counterparts in Fig. 4b, as they have access to substantial convective heat dissipation by the winds. But nevertheless, even with the presence of substantial air convection, we still observe an averaged temperature difference of 35.4 °C for the entire 3 h measurement period between the two photonic structures. In addition, comparing with the pink paint sample, the ‘cold’ photonic structure shows a temperature that is 15.4 °C lower than the pink paint whereas the ‘hot’ photonic structure shows a temperature that is 20.1 °C hotter. And the ‘hot’ photonic structure is 7.7 °C hotter than the black paint. Those results further demonstrate the efficacy of photonic engineering in thermal management of colour objects with desired colour perception, in outdoor conditions even with strong convective heat dissipation.

## Discussion

To conclude, we have highlighted the mechanisms through which one can control the radiative thermal load of coloured objects. We report significantly large tunable range of radiative thermal load for objects with the same colour, as a result of both physical and physiological effects. And we show those mechanisms can be integrated into a single structure to achieve a large tunable range of radiative thermal load for a specific colour. While in our experiments the photonic structures have specular reflective surfaces, the theoretical analysis, and hence the overall concept, is applicable for either specular or diffusive surfaces. Our results indicate the significant opportunities for tailoring the radiative thermal load for thermal management of buildings, vehicles and clothing in outdoor conditions, without affecting the visual perception, and highlight the opportunities through which photonic engineering can be used for improving energy efficiency in a wide range of outdoor applications.

## Methods

### Spectrum to colour calculation

The colours perceived by the human eye result from the interplay between the sensitivity of the eye’s three cone cells, the spectral intensity of the light source, and the spectral reflection from the surface. The sensitivities of the three cone cells responsible for colour vision at short, middle, and long wavelengths (Fig. 1a) are numerically represented by three colour matching functions, $\bar x\left( \lambda \right)$, $\bar y\left( \lambda \right)$ and $\bar z\left( \lambda \right)$ (Supplementary Note 5), which were established from experiments on a standard observer by the International Commission on Illumination (CIE) in 1931^32^. The colour matching functions can be thought of as the spectral sensitivity curves of three linear light detectors yielding the CIE tristimulus values *X*, *Y* and *Z*:

7 $$X = 100\frac{{{\int} {I\left( \lambda \right)r\left( \lambda \right)\bar x\left( \lambda \right){\mathrm{d}}\lambda } }}{{{\int} {I\left( \lambda \right)\bar y\left( \lambda \right){\mathrm{d}}\lambda } }}$$

8 $$Y = 100\frac{{{\int} {I\left( \lambda \right)r\left( \lambda \right)\bar y\left( \lambda \right){\mathrm{d}}\lambda } }}{{{\int} {I\left( \lambda \right)\bar y\left( \lambda \right){\mathrm{d}}\lambda } }}$$

9 $$Z = 100\frac{{{\int} {I\left( \lambda \right)r\left( \lambda \right)\bar z\left( \lambda \right){\mathrm{d}}\lambda } }}{{{\int} {I\left( \lambda \right)\bar y\left( \lambda \right){\mathrm{d}}\lambda } }}$$

where *I*(*λ*) is the spectral power distribution of the illuminant. Here we use a standard D65 illumination (Supplementary Note 5) to represent the typical outdoor illumination condition. *r*(*λ*) is the spectral reflectivity of the outdoor structure. Assuming the object is opaque, then *r*(*λ*) can be related with spectral absorptivity *ε*(*λ*) by *r*(*λ*) = 1 − *ε*(*λ*). The calculated CIE tristimulus values *X*, *Y* and *Z* can be used to determine the lightness and chromaticity of the colour. The lightness of the colour is determined by the parameter *Y*. And the chromaticity of the colour is determined by the normalized parameters:

10 $$x = \frac{X}{{X + Y + Z}}$$

11 $$y = \frac{Y}{{X + Y + Z}}$$

12 $$z = \frac{Z}{{X + Y + Z}}$$

The normalized parameters *x* and *y* are used to find the corresponding chromaticity in CIE 1931 colour space (Supplementary Note 5). One can also find the corresponding colour in a commonly used and perceptually uniform CIE LAB colour space, where ‘*L*’ represents the lightness, ‘*a*’ represents redness and greenness and ‘*b*’ represents yellowness and blueness. The combination of *a* and *b* determines colour chromaticity, which includes the relative saturation, or chroma $C_{{{ab}}} = \sqrt {a^2 + b^2}$, as well as the hue of the colour $h_{{ab}} = {\mathrm{arctan}}\left( {b/a} \right)$, as discussed in the main text. Converting *X*, *Y*, *Z* to *L*, *a*, *b* values can be done using the following equations^31^:

13 $$L = 116 \ f\left( {Y/Y_n} \right) - 16$$

14 $$a = 500\left[ {f\left( {X/X_n} \right) - f\left( {Y/Y_n} \right)} \right]$$

15 $$b = 200\left[ {f\left( {Y/Y_n} \right) - f\left( {Z/Z_n} \right)} \right]$$

where *X*_*n*_, *Y*_*n*_, *Z*_*n*_ are the tristimulus value of a reference white object, and:

16 $$f\left( s \right) = s^{1/3}\quad {\mathrm{if}}\quad s > \left( {24/116} \right)^3$$

17 $$f\left( s \right) = \left( {841/108} \right)s + 16/116\quad {\mathrm{if}}\quad s \le \left( {24/116} \right)^3$$

### Metamerism spectra calculation

We calculate the reflection spectra of a given colour with defined *L*_0_, *a*_0_ and *b*_0_ that provide the maximum and minimum net thermal load *P*_net_ by solving a constrained optimization problem. Given a reflection spectrum *r*(*λ*), its colour can be characterized by its *L*, *a*, *b* parameters as calculated with Eqs. (7)–(15). To characterize the thermal variability of a given colour, we denote the spectrum with the maximum and minimum thermal load as *r*_hot_(*λ*) and *r*_cold_(*λ*), respectively, such that

18 $$r_{{\mathrm{hot}}}\left( \lambda \right) = \begin{array}{*{20}{c}} { {\mathrm{arg}}\,{\mathrm{max}}} \\ {r\left( \lambda \right)} \end{array}P_{{\mathrm{net}}}\left( {r\left( \lambda \right)} \right)$$

19 $$r_{{\mathrm{cold}}}\left( \lambda \right) = \begin{array}{*{20}{c}} { {\mathrm{arg}}\,{\mathrm{min}}} \\ {r\left( \lambda \right)} \end{array}P_{{\mathrm{net}}}\left( {r\left( \lambda \right)} \right)$$

where *P*_net_ is defined in Eqn. (1). Meanwhile, for *r*_hot_(*λ*) and *r*_cold_(*λ*) to produce the same colour, they must satisfy the constraint, such that the colour difference with respect to the reference colour *L*_0_, *a*_0_ and *b*_0_, defined as

20 $${\mathrm{\Delta }}E\left( {r\left( \lambda \right)} \right) = \sqrt {\left( {L\left( {r\left( \lambda \right)} \right) - L_0} \right)^2 + \left( {a\left( {r\left( \lambda \right)} \right) - a_0} \right)^2 + \left( {b\left( {r\left( \lambda \right)} \right) - b_0} \right)^2}$$

is within 0.1 for both *r*_hot_(*λ*) and *r*_cold_(*λ*). The result from the optimization in Eqns. (22) and (23) give the reflection spectra that exhibit the same perceived colour but have either the maximum or minimum thermal load. This method was applied to compute the maximum and minimum thermal load associated with any colours, as presented in Fig. 2 and Supplementary Note 1.

### Photonic design

To design photonic structures with same colour but very different thermal loads, we developed an optimization scheme that implements memetic algorithm^33,34^ to optimize for the photonic structures, taking into account both thermal load and colour considerations. The optimization problem is a constrained optimization, such that the resulting reflection spectrum *r*(*λ*) of the photonic structures preserves the colour given by Eq. (20), and that its spectrum reaches maximum or minimum thermal load given by Eqs. (18) and (19). For consideration of both performance and fabrication, for each photonic structure we arrange three suitable constituent materials with desirable optical properties in both solar and thermal wavelength range, as described in the main text. In setting up the memetic algorithm optimization, we generate a population of structures that contains individual structures with randomly varying thicknesses, and each of their reflection spectra *r*(*λ*) can be computed efficiently using the method of impedance^35^ at near normal angle of incidence. According to the reflection spectra *r*(*λ*), we iteratively apply a set of standard evolutionary algorithm procedures to the population, such as crossover, mutation and reselection, which bypass stagnation in local minima. These operations are combined with intermittent local optimizations performed on selected individuals’ layer thicknesses using the quasi-Newton method to speed up the memetic algorithm convergence. At the end of the optimization, the structure that the population converges to contains the optimum thicknesses that result in a reflection spectrum *r*(*λ*) that reaches maximum/minimum thermal load while preserving the colour. This method was applied to design the photonic structures shown in Fig. 3, and can be extended to design photonic structures with other specific colours and thermal loads in general.

### Sample fabrication and characterization

The photonic structures were fabricated by electron beam deposition at Optiforms, on 500-um-thick, 100-mm-diameter, p-doped single-side-polished <100> crystalline silicon wafers, with resistivity 1–10 Ω cm (UniversityWafer 783). To block transmission in the wavelength range below silicon bandgap, the unpolished side of the wafers were coated with silver paste. In the wavelength range of 0.3−1.8 μm, the reflection spectra of the samples were characterized using a spectrophotometer (Agilent Cary 6000i) with an unpolarized light source and a high Lambertian reflectance standard (Labsphere SRS-99-020). A diffuse reflectance accessory (DRA 1800) with a 150-mm-diameter integrating sphere was used to collect both specular and diffuse components of reflection at an 8° angle of incidence. In the wavelength range of 4−25 μm, a Fourier transform infrared spectrometer (Thermo Scientific Nicolet 6700) with a diffuse gold integrating sphere (PIKE Technologies) was used to characterize the samples. The absorptivity/emissivity spectra were obtained by subtracting the reflectance from unity. A scanning electron microscope (Nova FEI 450) was used to image the cross sections of the stacks in Fig. 3a, b.

### Outdoor temperature measurements

The two photonic structures and reference paint samples were tested on a flat building roof (Fig. 4a) in Stanford, CA, in November 2017. The experimental apparatus for temperature measurements consist of an aluminized Mylar-coated wooden frame. A clear acrylic box with the top side open is joined and sealed to the underside of the wooden frame’s top surface. A polystyrene pedestal covered by aluminized Mylar is glued on the acrylic box. In the experiment of Fig. 4b, the top aperture of the wooden frame is covered with infrared transparent low-density polyethylene film to reduce the air convection effect. In the experiment of Fig. 4c, the top aperture of the wooden frame is left open to mimic the typical outdoor conditions in the presence of convective airflow cooling. The back surfaces of the samples are instrumented with an adhesive resistance temperature detector sensor with ±0.15 °C accuracy mounted on the centre of the structures, connected to a data logger (Omega OM-CP-OCTRTD). On the days that testing occurred, the sun’s peak elevation was around 35° above the horizon, whereas the apparatus containing the samples were placed on a platform tilted 55° toward the south. Thus, at maximum solar irradiance sunlight is near-normally incident on the samples. During the measurement, a pyranometer (Kipp & Zonen CMP6) and a data logger rated to a directional error of ±20 Wm^−2^ were used to record the solar irradiance incident on the samples. The pyranometer was placed on the same tilted platform as the apparatuses. An air temperature resistance temperature detector probe with ±0.15 °C accuracy is used to measure the ambient air temperature. The probe is mounted behind the platform, with free airflow and without access to direct sunlight irradiance.

## Electronic supplementary material

Supplementary Information

## Data Availability

The data that support the findings of this study are available from the corresponding author upon reasonable request.
