# Supplementary material for: Photonic thermal management of coloured objects
Source: Nat Commun. 2018 Oct 12;9:4240. doi: 10.1038/s41467-018-06535-0 (PMC6185958; doi:10.1038/s41467-018-06535-0)
Supplement: Supplementary file 1 — Supplementary Information [file 41467_2018_6535_MOESM1_ESM.pdf]

Supplementary Information

Photonic Thermal Management of Coloured Objects

Li et al.

Department of Electrical Engineering, Ginzton Laboratory, Stanford University, Stanford, California 94305, USA

Supplementary Note 1: Tunable range of radiative thermal load for the entire colour space.

We compute the tunable range of radiative thermal load over the entire colour space. First, Supplementary Figure 1 shows the computed entire achievable 3D colour gamut from a surface with arbitrary reflection spectrum $r(\lambda)$, under D65 illumination. This 3D colour gamut is plotted in a CIE LAB colour space, where the three axes are *L*, *a*, *b*, respectively.

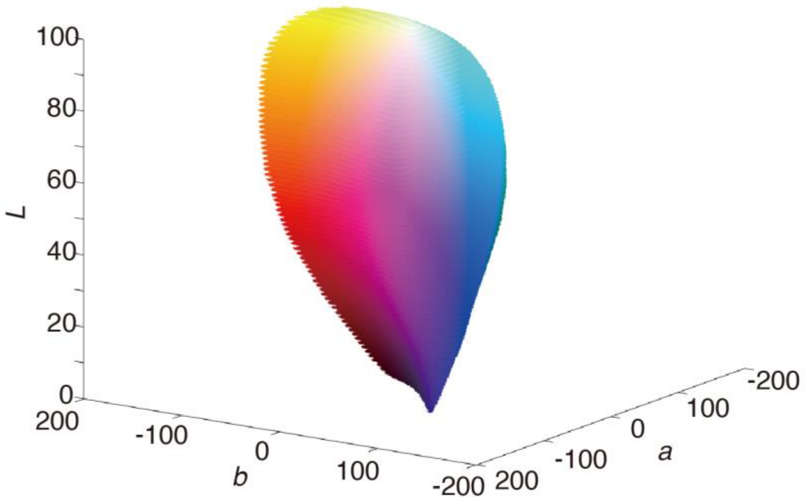

Supplementary Figure 1. Computed 3D colour gamut from a surface with arbitrary reflection spectrum under D65 illumination, plotted in a LAB colour space.

We now present the tunable range of radiative thermal load over this colour gamut. Supplementary Figure 2 shows the achievable colour space at 9 different lightness levels (*L* = 10, 20, 30, 40, 50, 60, 70, 80, 90), as well as the tunable range, as shown by the contour plot imposed on the colour space. At each lightness level, there is a defined colour range that can be achieved, corresponding to the McAdam limits[1,2]. At high lightness level, for example *L* = 90 (Supplementary Figure 2a), the achievable colour range mostly falls in the quadrant with negative *a* and positive *b* values, giving yellow and green colours. As the lightness *L* decreases (Supplementary Figure 2a-i), the colour range gradually shifts to the quadrant with positive *a* and

negative *b* values, giving blue and red colours (Supplementary Figure 2i).

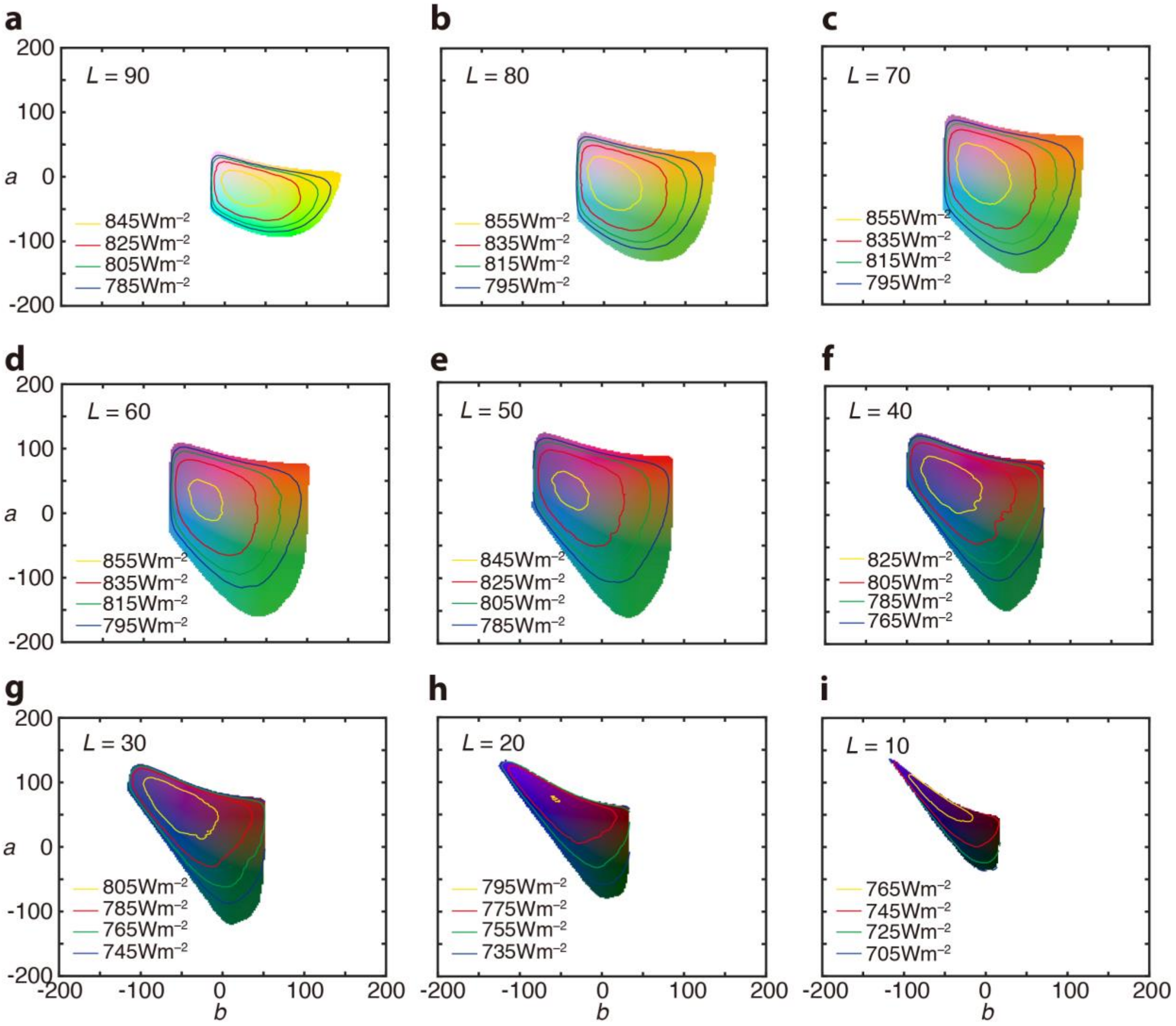

Supplementary Figure 2. Computed colour range and tunable range of radiative thermal load at different lightness level in a LAB colour space.

At each lightness level, we show in Supplementary Figure 2 the tunable range of radiative thermal load as contour plots. We observe that the tunable range increases as we move from the outer boundary of the achievable colour range into the center. In addition, the largest tunable range occurs at L = 80 (Supplementary Figure 2b) and L = 70 (Supplementary Figure 2c). As lightness L increases to L = 90 (Supplementary Figure 2a) or decreases to lower values (Supplementary Figure 2d-i), the tunable range gradually decreases. In the entire colour space,

the tunable range varies from 680 to 866 Wm^{-2}.

Supplementary Note 2: Temperature calculation under a typical outdoor condition

For the calculations performed in Fig. 2e in the main text, we are considering the possible temperature ranges for a variety of colours, in a typical outdoor condition. The h_c value (12 $Wm^{-2}\cdot K^{-1}$) we pick here assumes a typical rooftop scenario, which is in a similar situation as our experiment (Fig. 4c in the main text). In this scenario, the parasitic heat loss is dominated by forced convection[3] [pg. 309, Lienhard, J. H. & Lienhard, J. H. *A Heat Transfer Textbook*. (Dover Publications, Inc, 2011)]. Here the heat transfer coefficient of 12 $Wm^{-2}\cdot K^{-1}$ corresponds to a typical wind speed of 1 $m\cdot s^{-1}$, and a Nusselt number of 47, for a 4" wafer. The physical properties of air are chosen as viscosity = 1.58×10^{-5} $m^2\cdot s^{-1}$, diffusivity = 2.21×10^{-5} $m^2\cdot s^{-1}$.

Supplementary Note 3: Heat transfer analysis of photonic structures and reference samples

Here, for the experiment demonstration shown in Fig. 4b in the main text, we provide the experimentally measured transmission spectrum of the polyethylene film, as shown in Supplementary Figure 3.

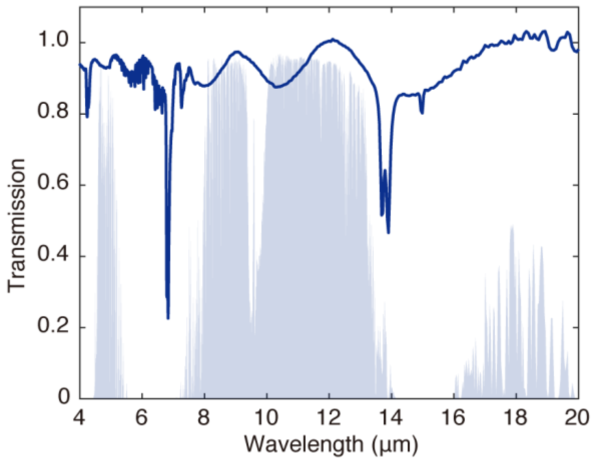

Supplementary Figure 3. Experimentally measured infrared transmission spectrum of polyethylene film. Atmosphere transmittance (light blue shaded area) is plotted for reference.

As can been seen, the polyethylene film is quite transparent in the entire infrared wavelength range, especially in the 8 to 13 μm wavelength range where the atmosphere is highly transparent. Therefore this polyethylene film can be used to suppress the non-radiative heat dissipation due to wind fluctuation, without affecting the radiative thermal load of the photonic structures in outdoor conditions. The use of the polyethylene film therefore enables us to experimentally demonstrate the large variation in the radiative thermal load for structures with the same colour.

We now model steady-state temperatures of the photonic structures and reference samples by balancing P_{net} (Eq. 1 in the main text) with non-radiative heat loss $P_{\text{cond+conv}} = h_{\text{c}}\left(T - T_{\text{amb}}\right)$, where h_{c} is the combined non-radiative heat coefficient taking into account both conduction and convection. And we compare them to the experimentally measured temperatures (Fig. 4b and c in the main text). For the modeling, we input the experimentally derived absorption/emission data of the photonic structures and reference samples, the AM1.5 spectrum weighed to the measured solar irradiance, and use a model of atmosphere transmittance MODTRAN5 to account for the atmosphere in the infrared for a clear sky at Stanford during the measurement days[4].

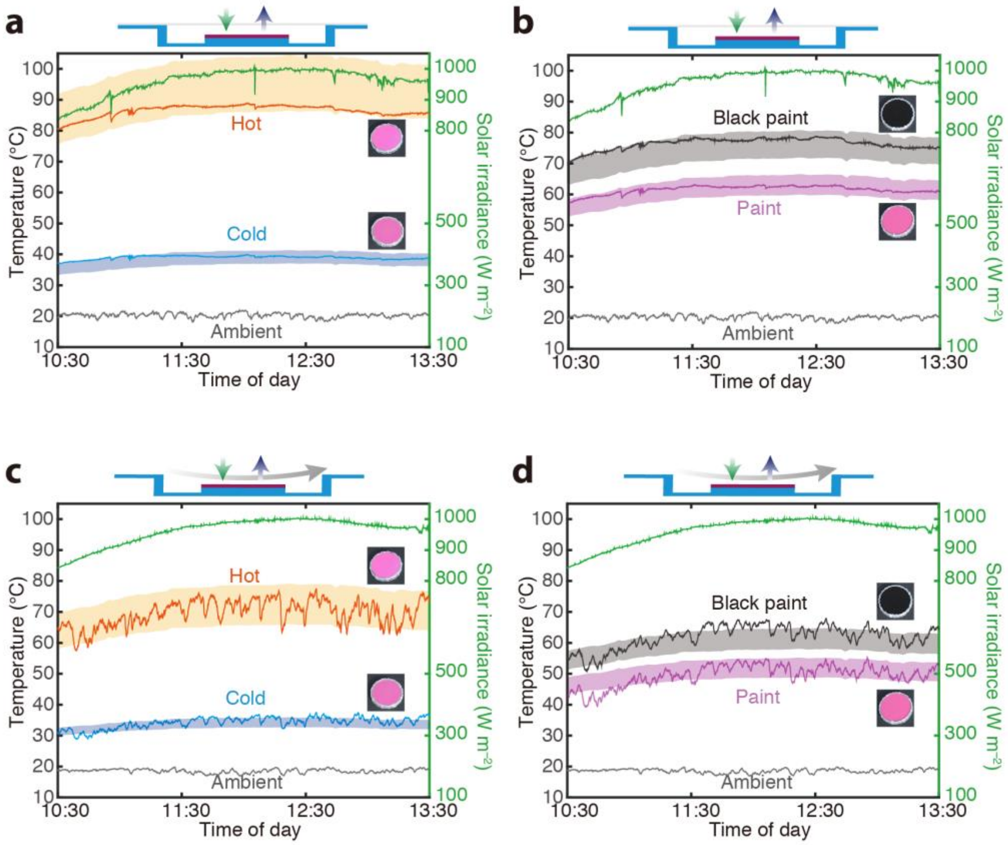

Supplementary Figure 4. Modeling of steady-state temperatures of photonic structures and reference paint samples. a, b. For the experiments with polyethylene cover (Fig. 4b in the main text), the modeled steady-state temperatures for the hot photonic structure, cold photonic structure, pink paint and black paint are shown as the light yellow, light blue, light purple and gray bands, respectively, for an h_c value range from 7 to 9.5 $Wm^{-2}\cdot K^{-1}$. The experimentally measured temperatures (Fig. 4b in the main text) of the hot photonic structure, cold photonic structure, pink paint and black paint are shown by the red, blue, light purple and black curves, respectively. **c, d.** For the experiments without polyethylene cover (Fig. 4c in the main text), the modeled steady-state temperatures for the hot photonic structure, cold photonic structure, pink paint and black paint are shown as the light yellow, light blue, light purple and gray bands, respectively, for an h_c value range from 11 to 15 $Wm^{-2}\cdot K^{-1}$. The experimentally measured temperatures (Fig. 4c in the main text) of the hot photonic structure, cold photonic structure, pink paint and black paint are shown by the red, blue, light purple and black curves, respectively.

Supplementary Figure 4a and b show for the experiment with polyethylene cover (Fig. 4b in the main text), the modeled temperature range for the photonic structures and reference samples. Supplementary Figure 4c and d show for the experiment without polyethylene cover (Fig. 4c in

the main text), the modeled temperature range for the photonic structures and reference samples. By comparing the experimental results and the thermal modeling results of the two experiments, we see that the non-radiative heat transfer coefficient h_c is indeed suppressed by this polyethylene cover. Therefore this polyethylene film can be used to reduce the non-radiative heat dissipation, without affecting the radiative thermal load, and provide a direct demonstration of the variation of the radiative thermal load.

Supplementary Note 4: The effect of radiative sky access on the thermal performance.

We perform an experimental study to further demonstrate the effect of sky access on the overall temperature performances. In this experiment, we replace the solar transparent and infrared transparent polyethylene cover with glass, which is solar transparent yet infrared opaque. In Supplementary Figure 5a, we plot the measured outdoor temperature of ‘hot’ photonic structure, ‘cold’ photonic structure and pink paint. For ease of comparison, in Supplementary Figure 5b we also plot the measured temperature of those three samples with solar transparent and infrared transparent polyethylene cover. With the glass cover, the temperatures of the paint and the ‘cold’ photonic structure both increase around 5°C degree, since both the ‘cold’ photonic structures and the paint sample have strong thermal emissivity. As the glass, which is opaque in the infrared wavelength range, blocks the radiative accesses to sky, the radiative cooling power P_{cooling} (Eq. 4 in the main text) is reduced. As a result, the total radiative thermal load P_{net} (Eq. 1 in the main text) increases. On the other hand, the temperature of the ‘hot’ photonic structure nearly stays the same due to its strongly suppressed thermal emissivity through photonic engineering. Therefore, the effect of sky access on the ‘hot’ photonic structure is minimum.

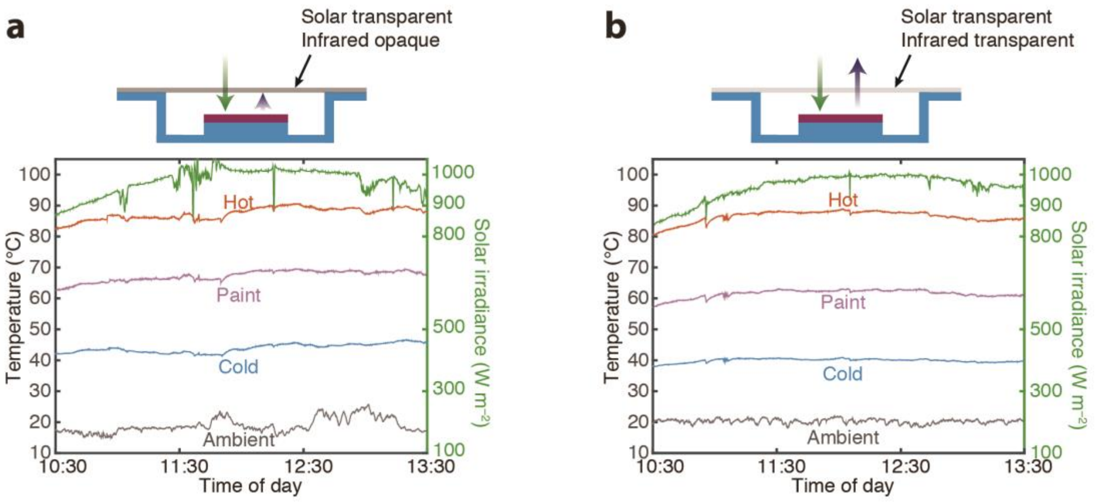

Supplementary Figure 5. The effect of radiative sky access on outdoor temperatures of photonic structures. a, With a solar transparent but infrared opaque glass cover, the experimentally measured temperatures of the hot photonic structure, cold photonic structure and pink paint are shown by the red, blue and light purple curves, respectively. **b,** With a solar transparent and infrared transparent polyethylene cover (Fig. 4b in the main text), the experimentally measured temperatures of the hot photonic structure, cold photonic structure, and pink paint are shown by the red, blue, and light purple curves, respectively.

Supplementary Note 5: Colour matching functions, D65 standard illumination, and CIE 1931 diagram

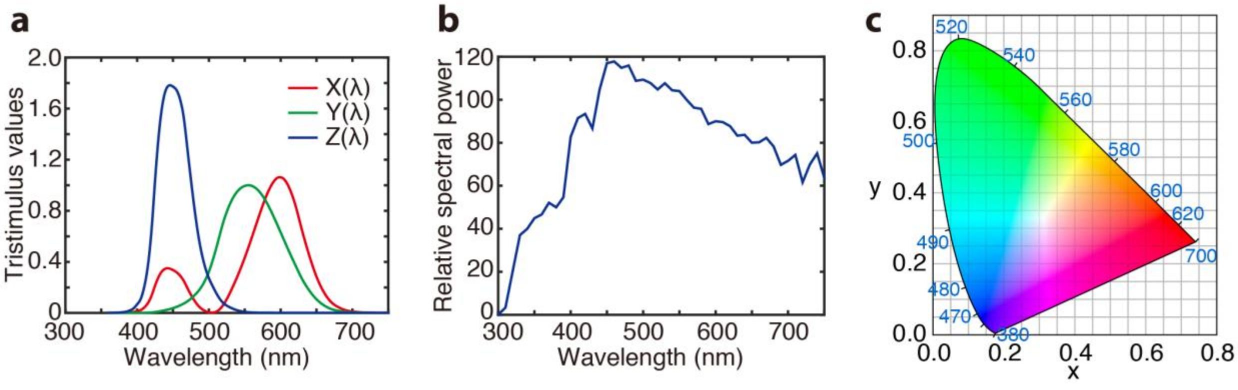

Supplementary Figure 6. a. CIE colour matching functions[5] **b.** Standard D65 light spectrum for representation of typical outdoor illumination conditions. **c.** CIE 1931 colour space.

Supplementary References

1. MacAdam, D. L. Maximum Visual Efficiency of Colored Materials. *J. Opt. Soc. Am.* **25,** 361–367 (1935).
2. MacAdam, D. L. The Theory of the Maximum Visual Efficiency of Colored Materials. *J. Opt. Soc. Am.* **25,** 249 (1935).
3. Lienhard, J. H. & Lienhard, J. H. *A heat transfer textbook*. (Dover Publications, 2011).
4. Berk, A. *et al.* MODTRAN5: 2006 update. in *Proceedings of SPIE* **6233,** 62331F–62331F–8 (2006).
5. Fairman, H. S., Brill, M. H. & Hemmendinger, H. How the CIE 1931 Color-Matching Functions Were Derived from Wright–Guild Data. *Col Res Appl* **22,** 11–23 (1997).
